# Commentary: Endoscopy-assisted surgical management for a giant axillary cystic lymphangioma: a case report

**DOI:** 10.3389/fsurg.2026.1783070

**Published:** 2026-03-12

**Authors:** Vilma S. Semini, Paolo Gasparella, Stephan Spendel, Emir Q. Haxhija

**Affiliations:** 1 Division of Plastic, Aesthetic and Reconstructive Surgery, Department of Surgery, Medical University of Graz, Graz, Austria; 2 Department of Pediatric and Adolescent Surgery, VASCERN VASCA Affiliated Partner Center, Medical University of Graz, Graz, Austria

**Keywords:** adolescent and young adult, children, classification of vascular anomalies, hazards & risk, ISSVA (International society for the study of vascular anomalies), lymphatic malformations (LM), minimally invasive surgeries (MIS), surgery

Dear Editor,

We read with great interest the article by Chen et al. entitled *Endoscopy-assisted surgical management for a giant axillary cystic lymphangioma: a case report* (1). The authors report, for the first time, that resection of a large axillary lymphangioma can be facilitated by endoscopic assistance. While the reported outcome is remarkable, several aspects of the manuscript merit clarification.

## Introduction

Lymphangiomas—or, more accurately, lymphatic malformations (LMs), as defined by the International Society for the Study of Vascular Anomalies (ISSVA) (2)—are not “uncommon congenital benign tumors with an incompletely understood pathogenesis”. Rather, they are well-characterized developmental anomalies of the lymphatic system, most commonly caused by somatic activating mutations in the *PIK3CA* gene (3). LMs may occur in any anatomical location and may be macrocystic, microcystic, or mixed in morphology. They may present as isolated lesions or in association with other vascular malformations (2, 3). These concepts have been firmly established in the literature for more than three decades and are also clearly outlined in the same reference cited by the authors to support their incorrect statement (4).

That reference additionally reports the correct incidence of LMs, ranging from 1 in 6,000 to 1 in 16,000 individuals (4), which differs substantially from the figures cited by Chen et al. (1), which are consistent with the incidence of LMs of the mesentery.

The patient described by Chen et al. was a 17-year-old female who had previously undergone “sclerotherapy for a right facial hemangioma at the age of 8 years”. From the perspective of the ISSVA classification, this statement is difficult to interpret, as sclerotherapy in an 8-year-old patient would typically be performed for a venous malformation rather than for a hemangioma.

## Case presentation and diagnosis

The authors describe a large, unilocular cyst located in the left axilla containing clear lymphatic fluid. In our experience, the sudden clinical presentation of a large LM during adolescence is most often attributable to acute intracystic hemorrhage into a pre-existing LM, frequently following minor trauma. It is highly unusual for a slender adolescent, such as the patient depicted in the figures, not to have noticed a large axillary LM earlier, as these lesions generally grow proportionally with the child unless accelerated by infection or injury. As no evidence of intracystic hemorrhage is visible in the published images, the pathophysiological mechanism underlying the sudden appearance of this large axillary LM remains unclear.

In the previously published diagnostic pathway for patients with LMs, magnetic resonance imaging (MRI) is recommended following initial ultrasonographic evaluation (3). Computed tomography (CT) is indicated only for assessment of osseous involvement when such pathology is suspected on MRI. Against this background, it is difficult to understand why a 17-year-old female patient underwent two chest CT examinations for evaluation of an axillary LM, particularly after ultrasonography had already demonstrated “a well-defined, irregular anechoic area with multiple septations and no significant internal vascularity, suggestive of a lymphatic origin”. Furthermore, it remains unclear why MRI was not performed at the 6-month postoperative follow-up, especially given that the patient had already undergone preoperative MRI. The use of chest CT for postoperative surveillance of an axillary LM unnecessarily increases exposure to ionizing radiation, while providing inferior soft-tissue contrast compared with MRI, which is clearly the preferred modality in this clinical context.

## Surgical technique

Axillary LMs are a pathology with which we are well acquainted (5). We have performed numerous resections of giant axillary LMs, frequently requiring meticulous neurovascular dissection. These lesions are characterized by thin walls and require careful handling to achieve complete and preferably intact excision. Uncontrolled disruption of cyst walls may result in incomplete resection and recurrence, which, although undesirable, is considerably less harmful than injury to the brachial plexus or axillary vessels. Recurrences, when clinically relevant, can also be managed with percutaneous sclerotherapy, particularly as LMs are not neoplastic. In many institutions, sclerotherapy represents the first-line treatment for unilocular lymphatic cysts such as the one reported by Chen et al.

During open resection, we typically bluntly dissect cyst walls from surrounding tissues and use bipolar cautery when required. Even when vital structures are circumferentially encased, they can be safely freed using blunt dissection techniques (5). Large cysts can also be removed through relatively small skin incisions, as they prolapse through the incision once adequately mobilized (6).

In contrast, we consider the use of hook cautery under endoscopic camera guidance, as reported by Chen et al., to be potentially hazardous. We therefore understand the authors' need to convert to open surgery to perform meticulous dissection from the axillary nerves and vessels. A fundamental principle of safe endoscopic surgery is fixation of the port and the instrument through a rigid structure that serves as a hypomochlion, allowing precise one-handed instrument control. Standard endoscopic surgery is typically performed using two working instruments, one in each hand. The insertion of instruments through a modified surgical glove lacks a stable hypomochlion. Although an insufflation pressure of 12 mmHg may maintain glove distension, it does not provide sufficient stability for safe manipulation. As a result, two hands are required per instrument, necessitating three surgeons for control of one camera and two working instruments. This setup renders the procedure technically challenging and potentially unsafe.

## Discussion

While minimizing surgical scars is an important goal, the benefit of this modified subcutaneous endoscopic approach in the presented case remains unclear. The authors report aspiration of 300 mL of lymphatic fluid and illustrate collapsed cyst walls that were ultimately resected via open surgery in the deepest axillary region. Was not the endoscopic approach intended to facilitate dissection precisely in this area? We routinely perform such procedures using surgical loupes without limitations in visualization.

In conclusion, we congratulate the authors on the successful treatment of their patient and on their innovative approach. Nevertheless, in most institutions, a unilocular lymphatic cyst such as the one described would be treated with cyst aspiration followed by instillation of an appropriate sclerosing agent. We hope future studies will consistently apply the ISSVA classification of vascular anomalies, ensuring precise communication and a shared scientific language.
